# Supplementary material for: Hydrangea paniculata coumarins attenuate experimental membranous nephritis by bidirectional interactions with the gut microbiota
Source: Commun Biol. 2023 Nov 22;6:1189. doi: 10.1038/s42003-023-05581-9 (PMC10665342; doi:10.1038/s42003-023-05581-9)
Supplement: Supplementary file 1 — Supplementary Information [file 42003_2023_5581_MOESM1_ESM.pdf]

Supplementary methods

Light Microscopy

Paraffin-embedded kidney and colon sections which were stained with hematoxylin and eosin (HE) or Masson's Trichrome (Accustain, Sigma, St Louis, MO) were observed under light microscopy. Image analysis software (NDP Viewer 2; Hamamatsu Photonics, Tokyo, Japan) was used to further analyze the images of panoramic scanning. Histological quantitation analysis was examined by two independent observers who were blinded to the treatment grouping. The glomerular hypertrophy was assessed by measuring glomerular cross-sectional area of the whole Bowman's capsule which was introduced by previous publication [13]. Extracellular matrix (ECM) deposition in the mesangium was also investigated in glomerular cross sections by Masson staining and graded semiquantitatively on a scale of 0–4, where grade 0 indicates very weak or absent mesangial staining, and grades 1–4 indicate focally strong staining in, respectively, < 25%, 25–50, 50–75 or 75–100% of the glomerular tuft. Average ECM deposition scores were calculated for each animal group based on scores on the five-point scale and the corresponding numbers of glomeruli. A total of 20 randomly selected glomeruli were evaluated for each animal, and all fields for morphometric analysis were randomly selected.

Tubulointerstitial damage (infiltration, protein cast, fibrosis, tubular dilatation, or atrophy) was evaluated semiquantitatively as in previous investigations [13], in which the grading was done according to the extension of the damaged tubulointerstitial area in the renal cortex: 0, normal; grade 1, <10%; grade 2, 10% to 25%; grade 3, 25% to 50%; grade 4, 50% to 75%; and grade 5, 75% to 100%. The extension of the damage was evaluated selecting visually the injured areas in successive fields in the cortical and juxtamedullary areas of each biopsy.

Colon tissue damage was scored as the following criteria [14]: 0, no damage; 1, lymphoepithelial lesions; 2, focal ulceration or surface mucosal erosion; 3, broad mucosal damage involving deeper structures of the intestinal wall. Inflammatory cell infiltration score was evaluated according to the criteria below [14]: 0, few inflammatory cells in the lamina propria; 1, increased infiltration of inflammatory cells into the lamina propria; 2, the group of inflammatory cells infiltrating into the submucosa; 3, transmural infiltration of inflammatory cells. Then, the histological score was determined by combining the scores of tissue damage and inflammatory cell infiltration. Each section was calculated based on 5 randomly chosen fields.

Microbiome sample collection and analysis

Fecal samples from each animal were frozen at -80°C prior to DNA extraction. Total genomic DNA was extracted using DNA Extraction Kit following the manufacturer's instructions. Quality and quantity of DNA was verified with NanoDrop and agarose gel. For bacterial diversity analysis, V3-V4 variable regions of 16S rRNA genes was amplified with universal primers 343F and 798R (343 Forward: 5'-TACGGRAGGCAGCAG-3';798 Reverse: 5'-AGGGTATCTAATCCT-3')[17]. Raw

sequencing data were in FASTQ format. Paired-end reads were then preprocessed using Trimmomatic software[18] to detect and cut off ambiguous bases (N). It also cut off low quality sequences with average quality score below 20 using sliding window trimming approach. After trimming, paired-end reads were assembled using FLASH software[19]. Parameters of assembly were: 10bp of minimal overlapping, 200bp of maximum overlapping and 20% of maximum mismatch rate. Sequences were performed further denoising as follows: reads with ambiguous, homologous sequences or below 200bp were abandoned. Reads with 75% of bases above Q20 were retained. Then, reads with chimera were detected and removed. These two steps were achieved using QIIME software (version 1.8.0)[20]. Clean reads were subjected to primer sequences removal and clustering to generate operational taxonomic units (OTUs) using Vsearch software with 97% similarity cutoff[21]. The representative read of each OTU was selected using QIIME package. All representative reads were annotated and blasted against Silva database Version 123 (16s rDNA) using RDP classifier (confidence threshold was 70%)[22]. Chao1 and Shannon are used to indicate α-diversity, PCoA is used to indicate β-diversity.

Untargeted metabolomics and measurement of metabolites

The samples were analyzed by a 2.1 × 100 mm ACQUITY 1.8 μm HSS T3 using a Waters Acquity™ UPLC system equipped with a Waters Xevo™ G2 QTof MS (Milford, MA, USA). The metabolomic procedure including sample preparation, metabolite separation and detection, data preprocessing and statistical analysis for metabolite identification was performed following previous protocols with minor modifications [25]. The methods of chromatographic separation and mass spectrometry were described in detail as follow:

The UPLC analysis was performed with a Waters Acquity™ Ultra Performance LC system (Waters Corporation, Milford, MA, USA) equipped with a Waters Xevo™ G2 QTof MS (Waters MS Technologies, Manchester, UK). Chromatographic separation was carried out at 40 °C on an ACQUITY UPLC HSS T3 column (2.1 × 100 mm, 1.8 μm, UK). The mobile phase consisted of water (A) and acetonitrile (B), each containing 0.1% formic acid. The optimized UPLC elution conditions were: 0–1.0 min, 1.0% B; 1.0–12.0 min, 1.0–99.0% B; 12.0–14.0 min, 99.0–1.0% B and 14.0–15.0 min, 1.0% B. The flow rate was 0.40 ml/min. The autosampler was maintained at 4 °C. Every 1 μl sample solution was injected for each run.

Mass spectrometry was performed on a Xevo™ G2 QTof (Waters MS Technologies, Manchester, UK). The scan range was from 50 to 1200 m/z. For positive electrospray mode, the capillary and cone voltage were set at 3 kV and 30 V, respectively. The desolvation gas was set to 600 l/h at a temperature of 450 °C; the cone gas was set to 50 l/h and the source temperature was set to 110 °C. The mass spectrometry was operated in W optics mode with 12,000 resolution using dynamic range extension. The data acquisition rate was set to 0.1 s, with a 0.014 s interscan delay. Collision energy ramp was 20–30 V. All analyses were acquired using the lockspray to ensure accuracy and reproducibility. Leucine–enkephalin was used as the lockmass at a concentration

of 300 ng/ml and flow rate of 5 µl/min. Data were collected in continuum mode, the lockspray frequency was set at 10 s, and data were averaged over 10 scans. All the acquisition and analysis of data were controlled by Waters MassLynx v4.1 software. The mass data acquired were imported to Prognosis QI and Markerlynx XS (Waters Corporation, MA, USA) within the Masslynx software for peak detection and alignment. The resultant data matrices were introduced to the EZinfo 2.0 software (Waters Corporation, Milford, MA, USA). The metabolic pathways that the differential metabolites were involved in were enriched using the Kyoto Encyclopedia of Genes and Genomes pathway (KEGG) tool, and significant altered pathways were represented by bubble chart using on-line bioinformatics tool (https://cloud.oebiotech.com/task).

Supplementary data

Supplementary Figure S1. Base-line body weight and albuminuria among all the groups. *, P<0.05, ***, P<0.001. (n=10). Bar graphs are means ± SD

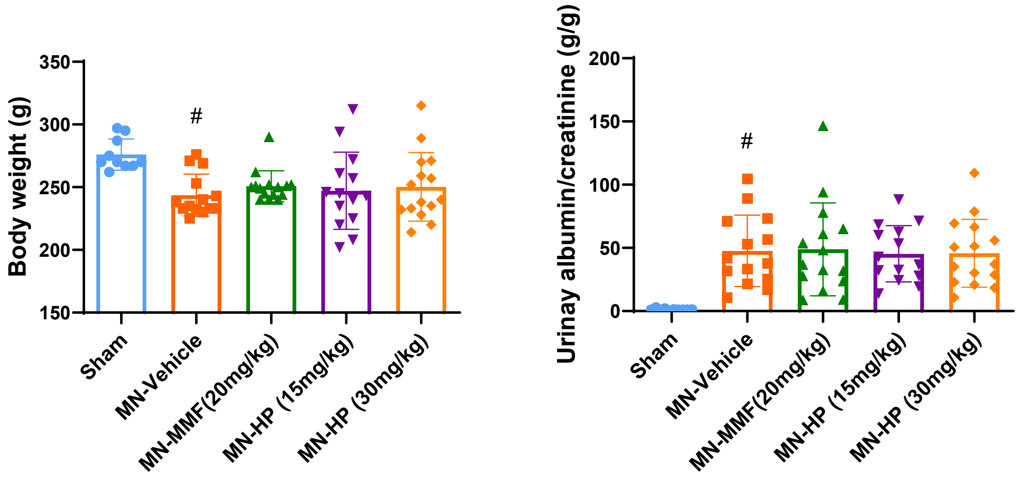

Supplementary Figure S2. Renal function manifested by albuminuria, serum NGAL, BUN, Scr, serum cholesterol, and kidney index. #, P<0.05, ##, P<0.01, ###, P<0.001 versus sham group; *, P<0.05, **, P<0.01, versus vehicle-treated group. (n=10). Bar graphs are means ± SD .

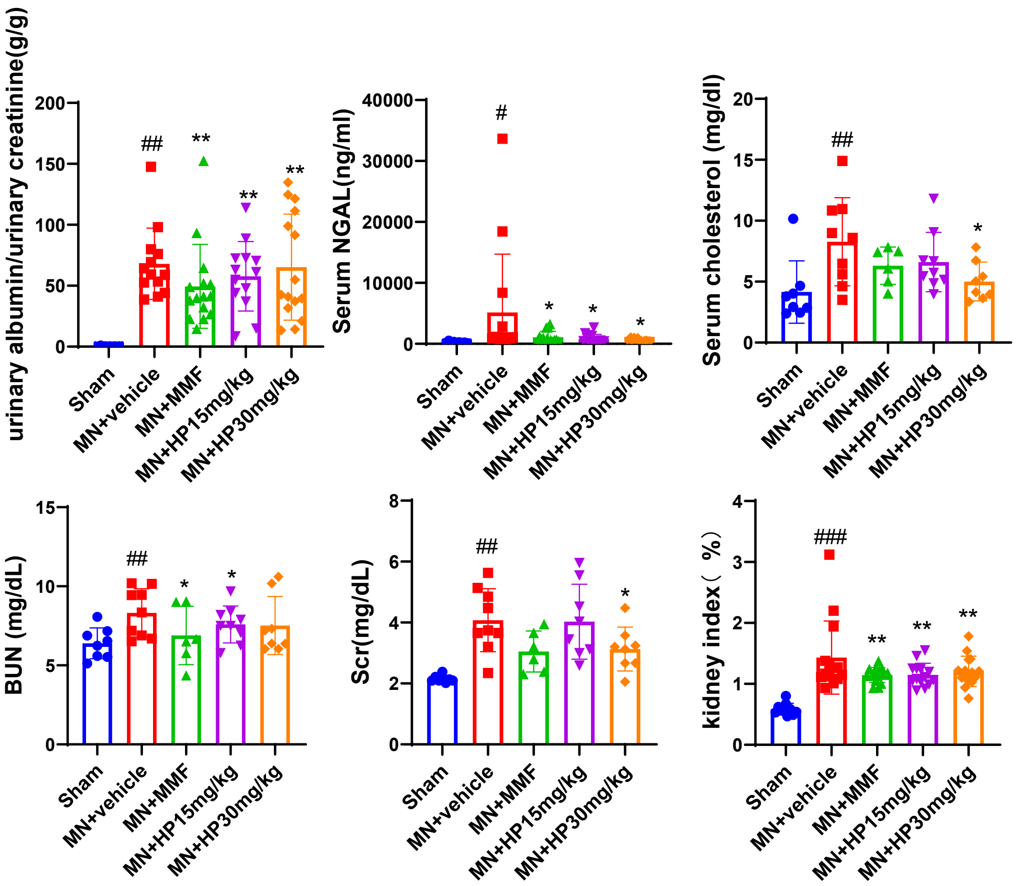

Supplementary Figure S3. Representative pathological photographs of light microscopy (H&E and Masson-stained) and electronic microscopy for glomeruli and renal interstitium; the blue circle refers to infiltration of inflammatory cells; Red arrows refers to protein cast and tubular dilatation; Black arrows refers to Masson dark staining with potential glomerulosclerosis; Yellow arrows refers to podocytes, which we can find there is almost no integral podocytes in MN model group treated by vehicle.

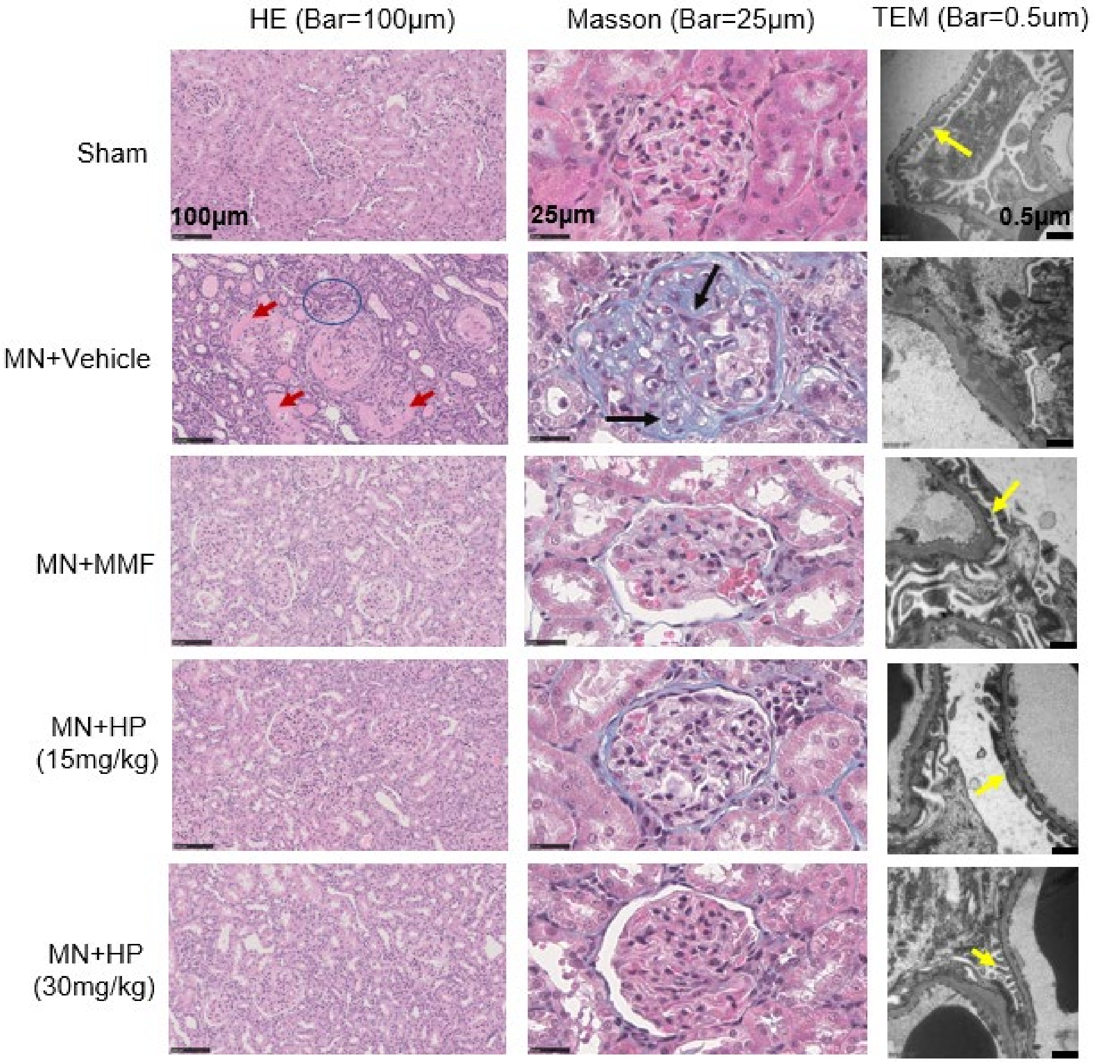

Supplementary Figure S4. Quantitative analysis of glomerular hypertrophy, glomerulosclerosis, tubulointerstitial damage, GBM thickness and podocyte loss. #, P<0.05, ##, P<0.01, ###, P<0.001 versus sham group; *, P<0.05, **, P<0.01, versus vehicle-treated group. (n=10). Bar graphs are means ± SD.

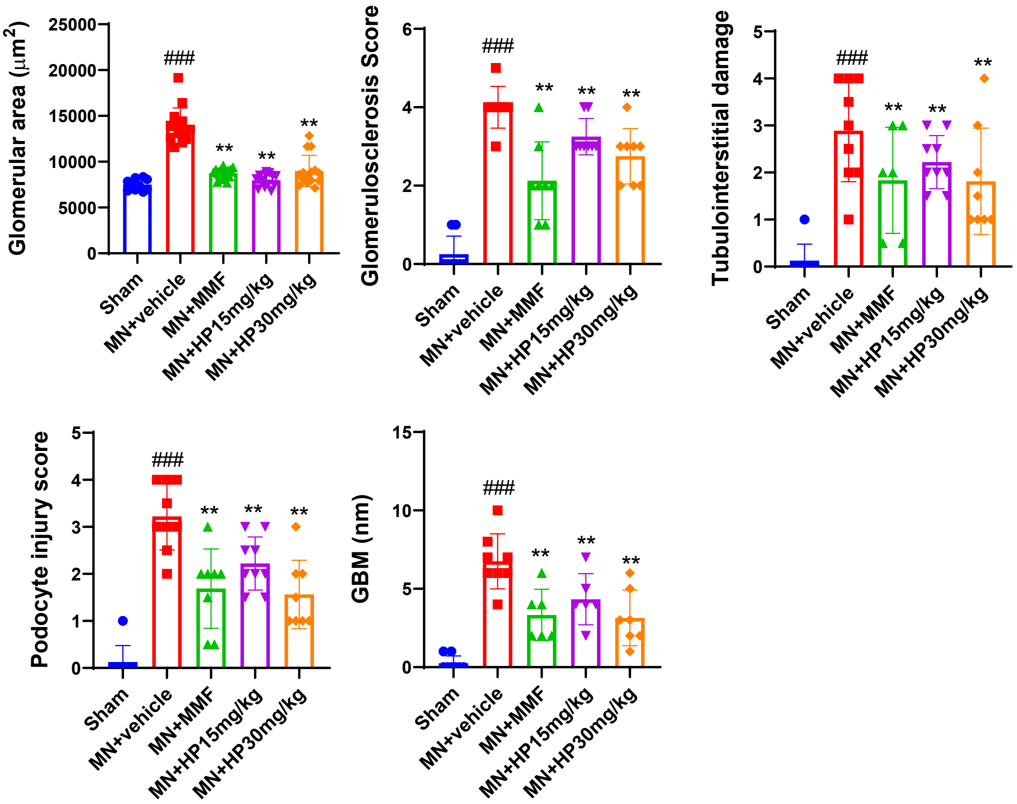

Supplementary Figure S5. CD68+ macrophage infiltration into the kidney tissues is attenuated by HP treatment. (n=10). Bar graphs are means ± SD.

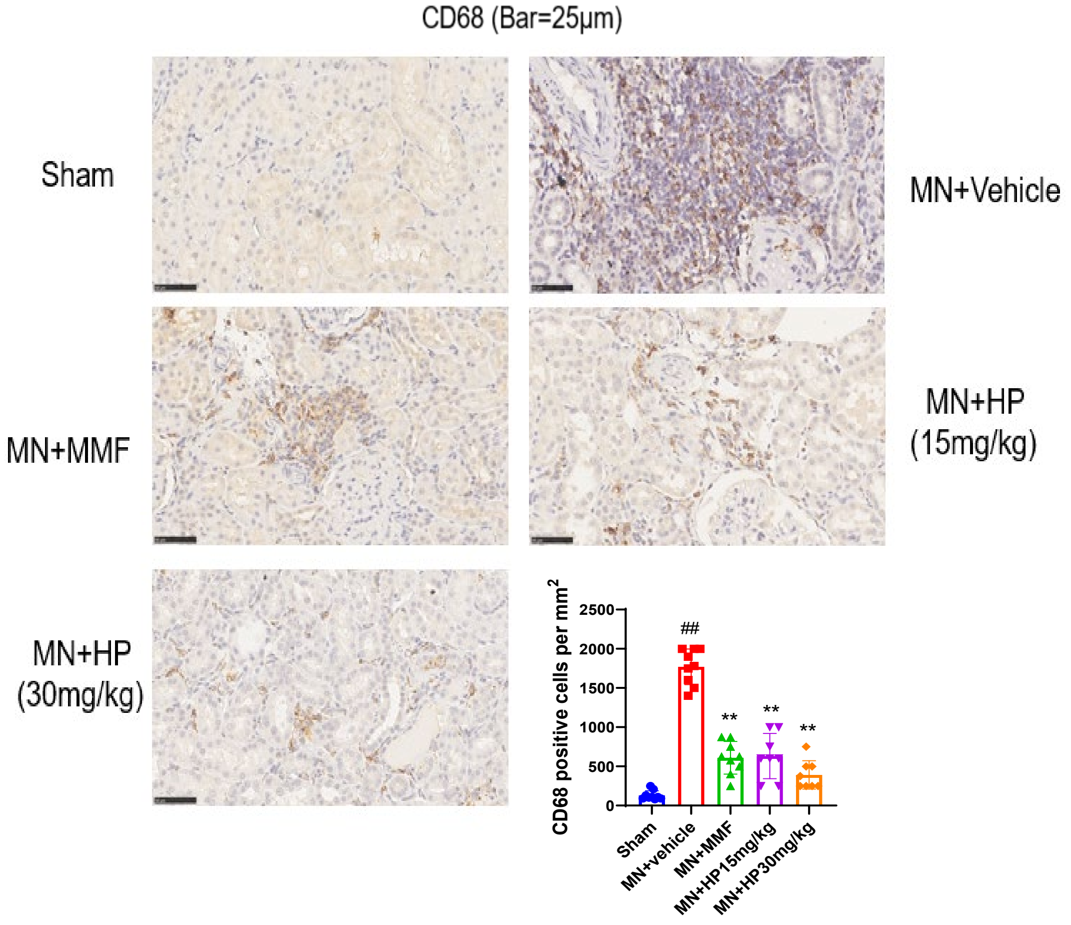

Supplementary Figure S6. HP treatment reverses the reduced richness of gut microbiota in c-BSA induced MN rats by increasing Chao1 and observed species (n=10). Bar graphs are means ± SD.

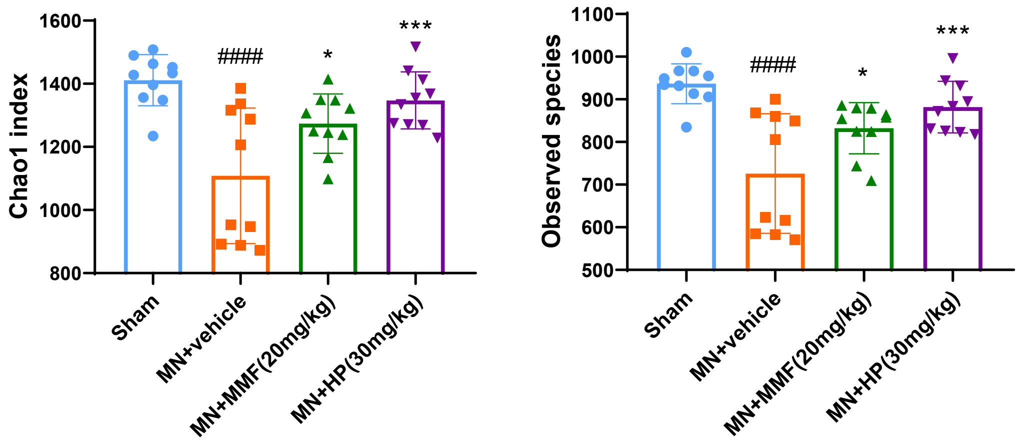

Supplementary Figure S7. Differential fecal metabolites between sham and MN-vehicle rats.

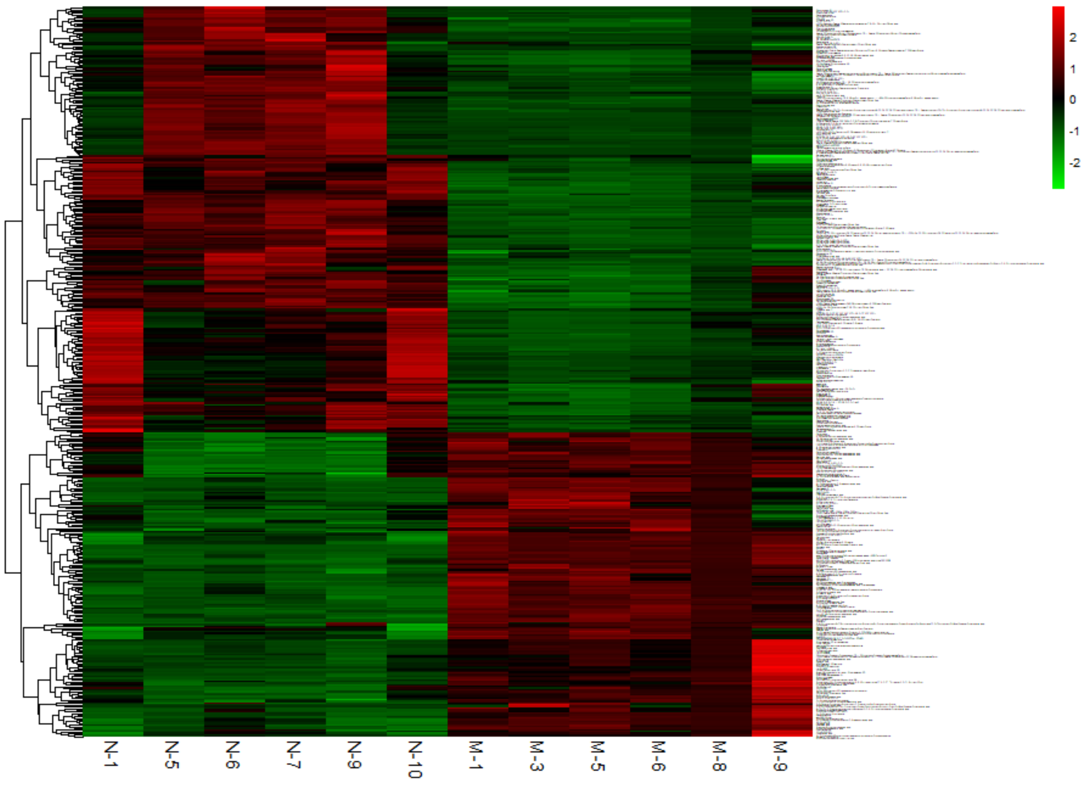

Supplementary Figure S8. Antibiotics administration depletes the abundance of gut bacterium effectively in mice measured by counting the bacterium colonies in streak culture using faeces from animals.

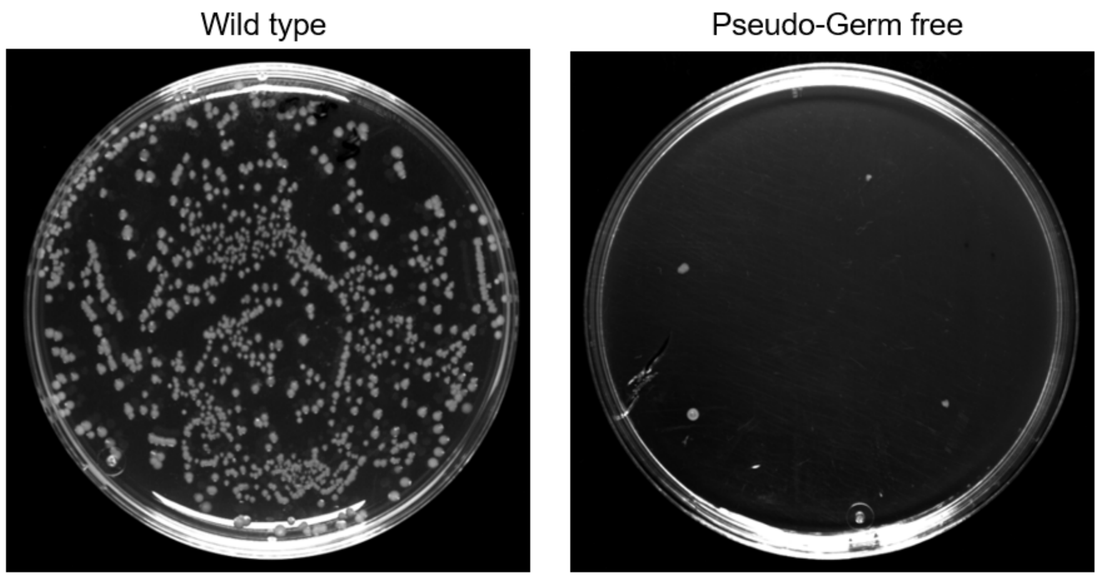

Supplementary Figure S9. 7-hydroxylcoumarin has higher anti-ROS capability than skimmin and apiosylskimmin in H_2O_2 induced ROS assay using rat kidney mesangial cells. (n=3). Bar graphs are means ± SD.

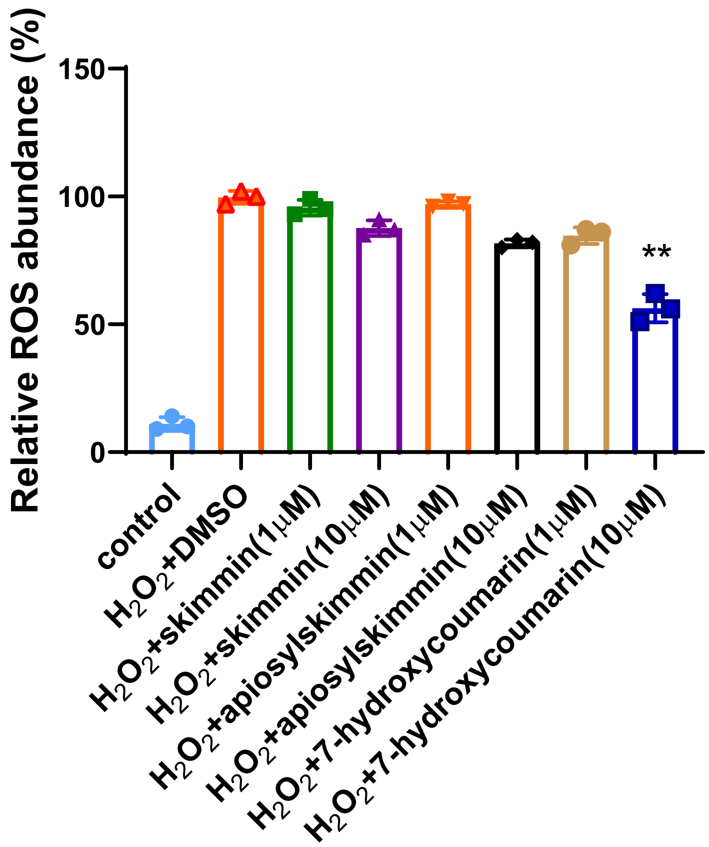

Supplementary Figure S10. HP treatment significantly reduces the conjugated

indole derivates and p-cresol glucuronide in feces of MN rats. (n=6). Bar graphs are means ± SD.

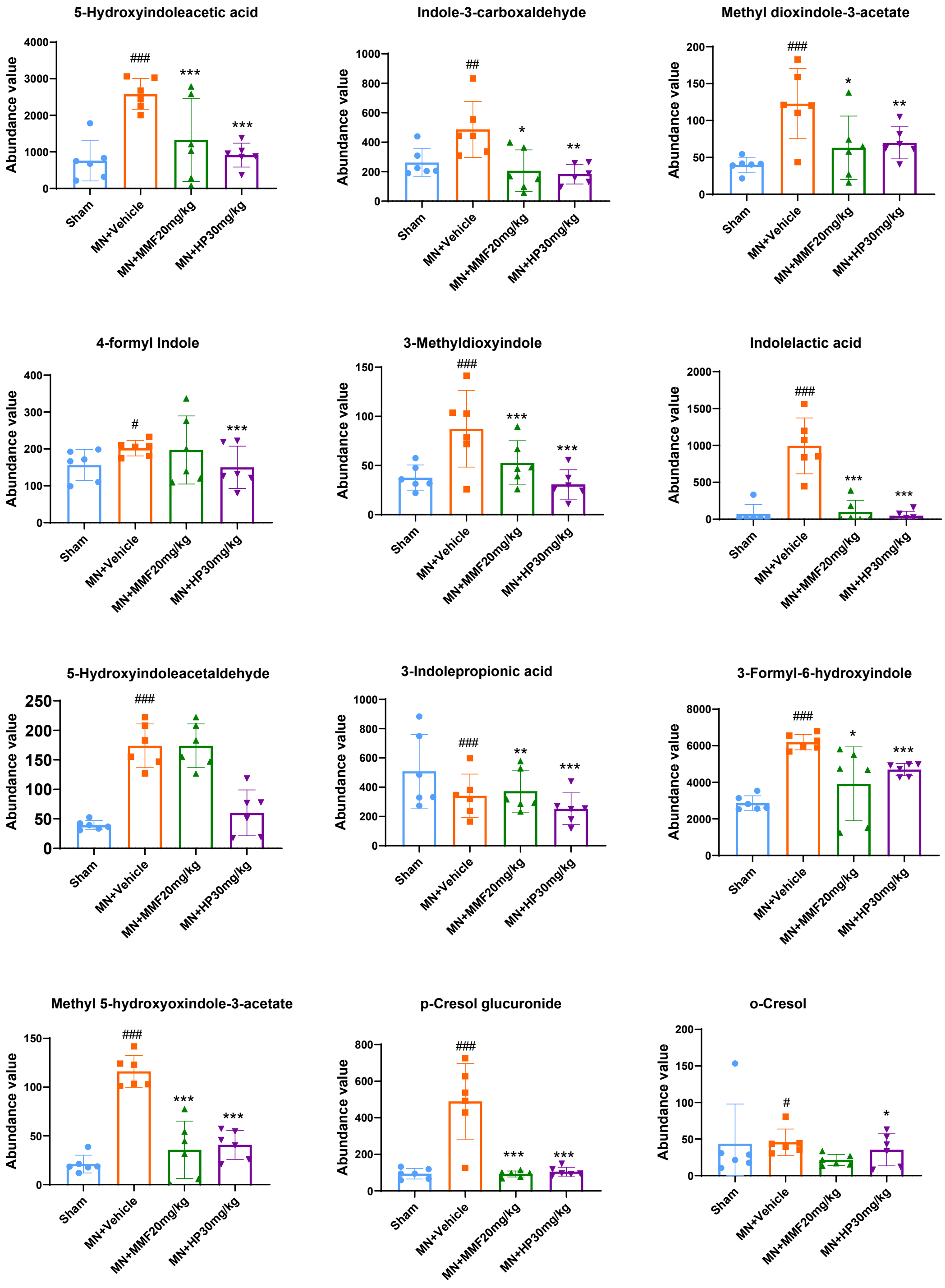

Supplementary Figure S11. High-performance liquid chromatography (HPLC)-based chemoprofile of coumarin derivates from HP. **a** HPLC profile at 318 nm; **b** HPLC profile at 280 nm. Standard skimmin and apiosylskimmin were purchased from Guilin Huiang Biochemistry Pharmaceutical Company Ltd (China).

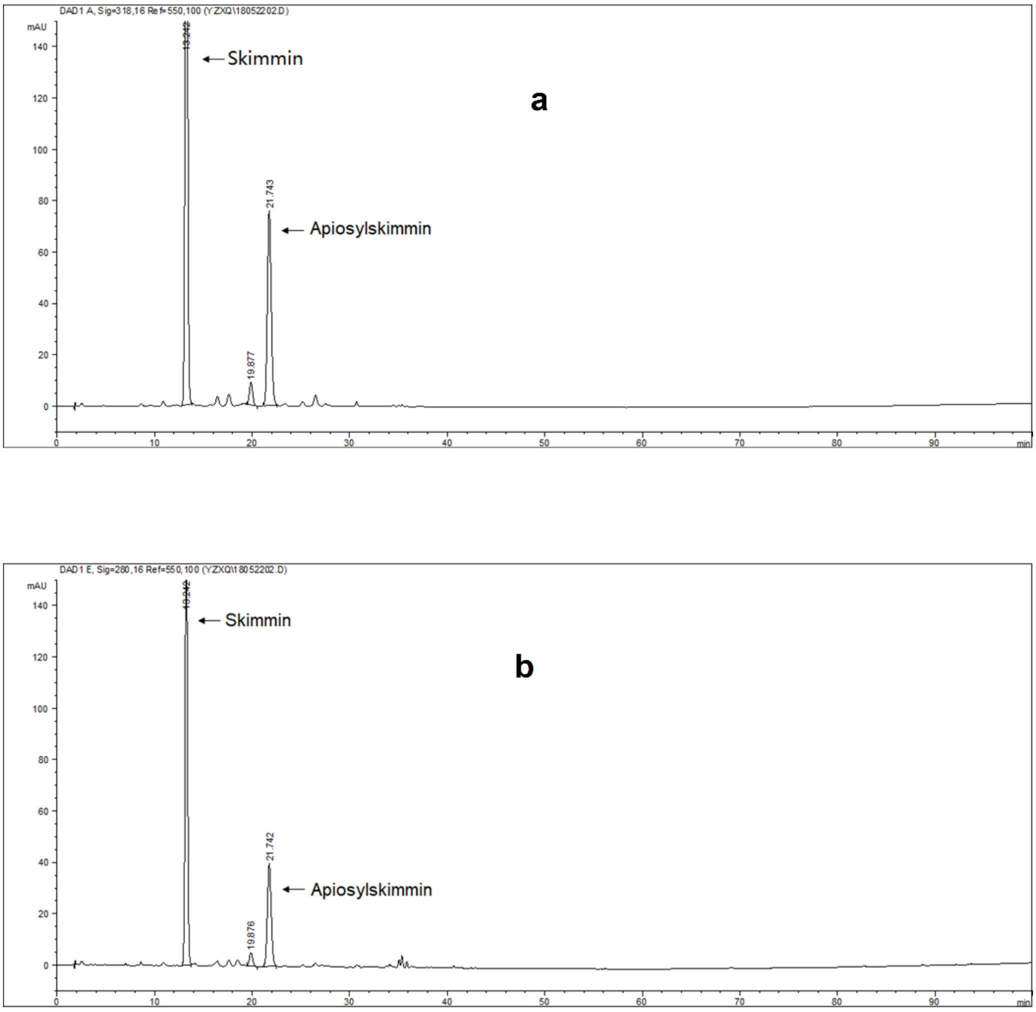

Supplementary Figure S12. Identification of isoelectric point (PI) of BSA and c-BSA using capillary Isoelectric Focusing.

BSA

c-BSA

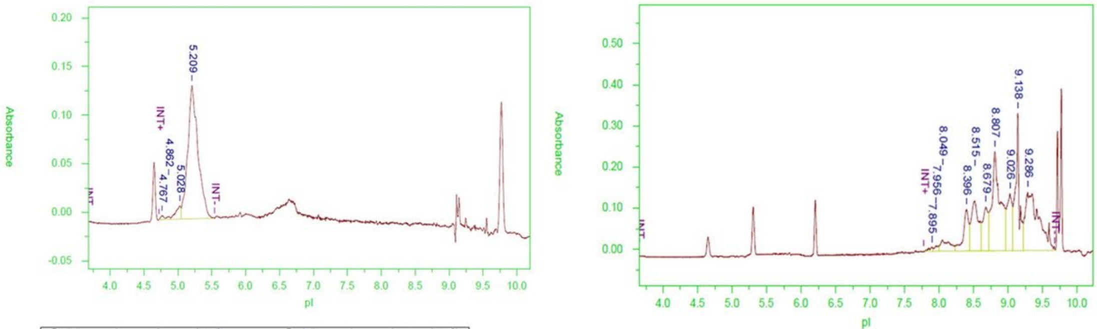

Peak #	pI	Area	Area %	Peak #	pI	Area	Area %
1	4.77	12528	0.815	3	5.03	72893	4.744
2	4.86	10081	0.656	4	5.21	1441133	93.785

Peak #	pI	Area	Area %	Peak #	pI	Area	Area %
1	7.90	38024	0.455	6	8.68	604333	7.234
2	7.96	54075	0.647	7	8.81	1980806	23.712
3	8.05	255852	3.063	8	9.03	731779	8.760
4	8.40	561897	6.726	9	9.14	1251189	14.978
5	8.52	890634	10.662	10	9.29	1985100	23.763

Supplementary Figure S13. Scheme of establishment of experimental models and drug administration.

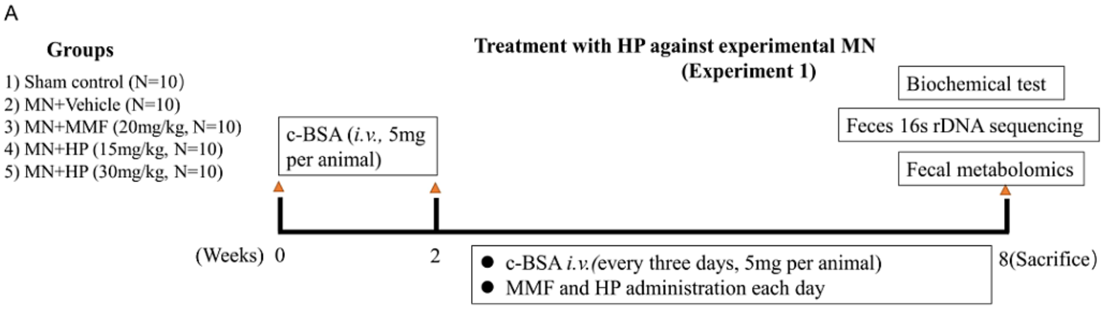

Supplementary Table S1 Primer sequence

Gene name	Primer sequence (from 5' to 3')	Gen bank accession number
IFN-γ forward	CAGGCCATCAGCAACAACAT	NM_138880
IFN-γ reverse	ATCTGTGGGTTGTTCACCTCG	
CXCL1 forward	CAGACAGTGGCAGGGATTCA	NM_030845
CXCL1 reverse	GGGGACACCCTTTAGCATCT	
CCL2 forward	AGCCAACTCTCACTGAAGC	NM_031530
CCL2 reverse	GTGAATGAGTAGCAGCAGGT	
TNF-α forward	ACCACGCTCTTCTGTCTACTG	NM_012675
TNF-α reverse	CTTGGTGGTTTGCTACGAC	
GM-CSF forward	ATACAAGCAGGGTCTACGGG	NM_053852
GM-CSF reverse	AGTCAGTTTCCGGGGTTGGA	
IL18 forward	GACCACTTTGGCAGACTTCAC	XM_039080947
IL18 reverse	GGGATTCGTTGGCTGTTCG	
IL13 forward	ATCACACAAGACCAGAAGACTTC	NM_053828
IL13 reverse	AACTGGGCTACTTCGATTTTGG	
IL9 forward	GTGACATACGTCCTTGCCTCT	NM_001105747
IL9 reverse	GATGGGTCGTCCTTCAGGTTTT	
IL17A forward	ATCCATGTGCCTGATGCTGTT	NM_001106897
IL17A reverse	AAAGTTATTGGCCTCGGCGT	
IL33 forward	GACCAGCTATCTCCCATCACT	NM_001014166
IL33 reverse	GGTCTTCTGTTGGGATCTTGTG	
IL22 forward	CAACCGCACCTTTATGCTGG	NM_001191988
IL22 reverse	ATCCTTGGCTTTGACTCCTCG	
IL6 forward	CCAGTTGCCTTCTTGGGACT	NM_012589
IL6 reverse	TGCCATTGCACAACTCTTTTC	
IL1α forward	GTGTTGCTGAAGGAGATTCCG	XM_039104245
IL1α reverse	AGCTGCGGATGTGAAGTAGT	
