# Supplementary material for: Hydrangea paniculata coumarins attenuate experimental membranous nephritis by bidirectional interactions with the gut microbiota
Source: Commun Biol. 2023 Nov 22;6:1189. doi: 10.1038/s42003-023-05581-9 (PMC10665342; doi:10.1038/s42003-023-05581-9)
Supplement: Supplementary file 3 — Description of Supplementary Materials [file 42003_2023_5581_MOESM3_ESM.docx]

**Description of Additional Supplementary Files**

**File name:** Supplementary Data 1

**Description:** The source data behind the figure 2J, K,L,M.

**File name:** Supplementary Data 2

**Description:** The source data of figure 2J, K,L,M, which are untargeted metabolomics data using rat faecal samples.
